# Pre-screening village tuberculosis notification rates and the yield of interferon-gamma release assay testing in a rural health-examination campaign in Xinjiang, China

**DOI:** 10.3389/fpubh.2026.1869406

**Published:** 2026-09-17

**Authors:** Weiyun He, Munirekezi Maimaitiyiming, Wei Xie, Fujin Liu, Yajuan Feng, Kunhong Jiang, Keng Lai

**Affiliations:** 1 State Key Laboratory of Respiratory Disease, Guangzhou Key Laboratory of Tuberculosis Research, Department of Tuberculosis Control and Management, Guangzhou Chest Hospital, Institute of Tuberculosis, Guangzhou Medical University, Guangzhou, Guangdong, China; 2 Disease Prevention and Control Center of Shufu County, Kashgar, Xinjiang, China

**Keywords:** annual health examination, interferon-gamma release assay, tuberculosis, tuberculosis infection, village-level surveillance, Xinjiang

## Abstract

**Background:**

Southern Xinjiang has a high tuberculosis (TB) burden, but the yield of interferon-gamma release assay (IGRA) testing within its annual rural health-examination program has not been quantified. We estimated IGRA positivity among adult campaign participants and assessed whether positivity increased with the village TB notification rate recorded before screening.

**Methods:**

This service-embedded cross-sectional study included adults who received IGRA testing during the annual health-examination campaign implemented across all 22 villages and farm units of Zhanmin Township in July–August 2024. Exclusion of 12 indeterminate results and two duplicate records left 3,742 unique adults. The principal exposure was the pre-screening average annual village TB notification rate (January 2021–June 2024). Logistic generalized estimating equations accounted for village clustering, adjusted for prespecified individual covariates, and used a Mancl–DeRouen small-sample covariance correction. Village-level regression, burden-group comparisons, and 1,000 village-cluster bootstrap resamples complemented the principal analysis.

**Results:**

Overall, 1,006 of 3,742 participants were IGRA positive (26.88%, 95% CI 25.49–28.33%); village-specific positivity ranged from 19.13 to 39.67%. High-burden villages contributed 39.56% of positive results while accounting for 33.27% of participants. Pooled positivity was 31.97% in high-burden villages, compared with 24.51 and 24.19% in low- and medium-burden villages. Each increase of 100 notifications per 100,000 was associated with 15.3% higher adjusted odds of positivity (OR 1.153, 95% CI 1.043–1.275; *p* = 0.006); the village-bootstrap estimate was similar. At village level, the corresponding increase in positivity was 2.92 percentage points (95% CI 1.18–4.67; *p* = 0.002). Male sex, ages 50–79 years, lower-income program category, known TB exposure, and previous TB were independently associated with positivity.

**Conclusion:**

IGRA positivity was common among campaign participants and increased with pre-screening village notification rates. Within the annual health-examination program, the continuous notification rate can be combined with individual risk factors to inform the scheduling of IGRA outreach and clinical assessment while maintaining access in every village. The 26.88% estimate pertains to participants and should not be interpreted as township prevalence.

## Introduction

Tuberculosis (TB) remains a major cause of preventable illness and death. The World Health Organization estimated that 10.7 million people developed TB in 2024 (1), while progress in reducing incidence remained short of the End TB Strategy milestones (2). Sustained incidence reduction requires both timely diagnosis of TB disease and prevention among people with *Mycobacterium tuberculosis* infection who are at increased risk of progression.

Approximately one quarter of the world’s population has been infected with *Mycobacterium tuberculosis* (3, 4), but infection is not evenly distributed. Population-based cohorts in rural China have documented marked differences in interferon-gamma release assay (IGRA) positivity by age and study site (5, 6). This heterogeneity matters operationally because IGRA testing requires phlebotomy, timely laboratory processing, and clinical follow-up. In high-burden southern Xinjiang, village-level infection data remain scarce. The expected yield of campaign-based testing and the usefulness of existing surveillance data for scheduling that testing are therefore uncertain.

IGRAs detect an immune response to *M. tuberculosis* antigens and are less affected by Bacille Calmette–Guérin vaccination than the tuberculin skin test. A positive result does not date infection, distinguish remote from recent infection, or exclude active TB (7). Reversion and conversion on repeat testing add further uncertainty (8). These constraints define both the study endpoint and the clinical pathway: IGRA positivity indicates immune sensitization, not active TB, and positive results require clinical assessment before preventive treatment is considered.

Systematic screening is most useful when the target population, diagnostic route, referral capacity, and expected yield are explicit (9). TB screening was incorporated into Xinjiang’s universal health-examination program in 2018 (10). Xinjiang Uyghur Autonomous Region has a high TB burden, with marked geographic heterogeneity in southern Xinjiang (11, 12). In Zhanmin Township, adult permanent residents may receive one free examination each year; chest radiography is the principal TB-screening item, and township staff refer people with abnormal radiographs or other TB suspicion to the county-designated TB hospital. Residents choose whether to attend, and recruitment uses continuing village-based mobilization. The campaign therefore provides program-based evidence on testing yield among participants rather than a probability-based estimate for all township adults.

Planning infection testing within this platform requires estimates of the yield among participants, its variation between villages, and whether pre-screening notification rates contain information beyond participant-level risk factors. We estimated IGRA positivity among adults tested during the 2024 Zhanmin campaign and examined its association with the average annual village TB notification rate recorded from January 2021 through June 2024. The continuous rate was the principal contextual exposure; tertiles were retained only as an operational summary. Individual risk factors were assessed in the same cluster-aware model to determine whether notification history added information beyond participant characteristics.

## Materials and methods

### Study design, setting, and routine examination platform

This service-embedded cross-sectional study was conducted across all 22 villages and farm units in Zhanmin Township, Shufu County, Kashgar Prefecture, Xinjiang, China. IGRA testing was conducted during July–August 2024 alongside the local annual health-examination program. The examination is available free of charge to adult permanent residents and includes chest radiography as the main TB-screening item (10). Township health-center staff coordinate referral of people with abnormal radiographs or other TB suspicion to the county-designated TB hospital for centralized diagnostic evaluation.

### Participants and analytic flow

The analytic cohort comprised adult permanent residents who attended the July–August campaign, provided written informed consent, and had an AIMTB record. The routine program used continuing village-based mobilization rather than a one-time named invitation list. The analytic source included every adult AIMTB record generated after consent; no investigator-selected subsample or additional within-village sampling was applied. The test export contained 3,756 records before analytic cleaning. Twelve indeterminate results were removed according to the AIMTB interpretation rules. The remaining 3,744 determinate records were checked by identifier and village linkage. Repeated identifiers were collapsed to one person-level record. Stable covariates agreed within each pair; if valid determinate AIMTB results differed, a positive result took precedence under the project’s deterministic any-positive reconciliation rule. This reduced the file by two records and produced a frozen cohort of 3,742 unique adults from 22 units, including 1,006 IGRA-positive and 2,736 IGRA-negative participants (Supplementary Figure S1). Core model variables were complete.

### Data sources and variable definitions

Trained staff used the examination record and a structured questionnaire to collect age, sex, ethnicity, education, occupation, diabetes status, known TB exposure, previous TB, and enrollment in the local lower-income or minimum-living-guarantee program. The lower-income category corresponded to household per capita annual income below RMB 5,292 under local program rules; continuous household income was not collected for this study. The standardized village ID field linked the participant, surveillance, and location files; free-text address descriptors were not used for analytic linkage (Supplementary Table S1). Village-level permanent-resident counts available from the administrative source referred to residents of all ages and were used only to describe program reach, not to define an adult sampling fraction.

The contextual exposure was the archived average annual TB case-notification rate per 100,000 permanent residents for January 2021–June 2024. The source surveillance file supplied one pre-calculated rate for each unit, derived from notified case counts and permanent-resident denominators; patient-level notification records were not used to reconstruct these values. June 2024 was the last complete pre-screening month. Rates were analyzed continuously, scaled per 100 notifications per 100,000. For operational summaries, the 22 rates were ranked into tertiles: low burden, ≤136.70 per 100,000; medium burden, >136.70–208.33; and high burden, >208.33. These cutoffs were data-derived planning categories rather than biological thresholds.

The latest official vector layer reflecting the current village-boundary configuration was not available. Figure 1 was therefore prepared as a thematic map using the project-provided Shufu County vector layer in the China Geodetic Coordinate System 2000 as the cartographic base. Village IDs and label positions were reconciled with the current village distribution and project administrative records. Twenty-one analytic units corresponded to distinct features in the available base layer. Study unit 20 was not represented by a distinct feature and is shown at its recorded location with a diamond; no missing boundary was inferred or constructed. Polygon or diamond fill encodes IGRA-positive prevalence or the pre-screening notification rate. The map is intended to display the geographic distribution of the observed village values and should not be interpreted as the latest legal administrative-boundary map or as a basis for formal adjacency analysis.

**Figure 1:**
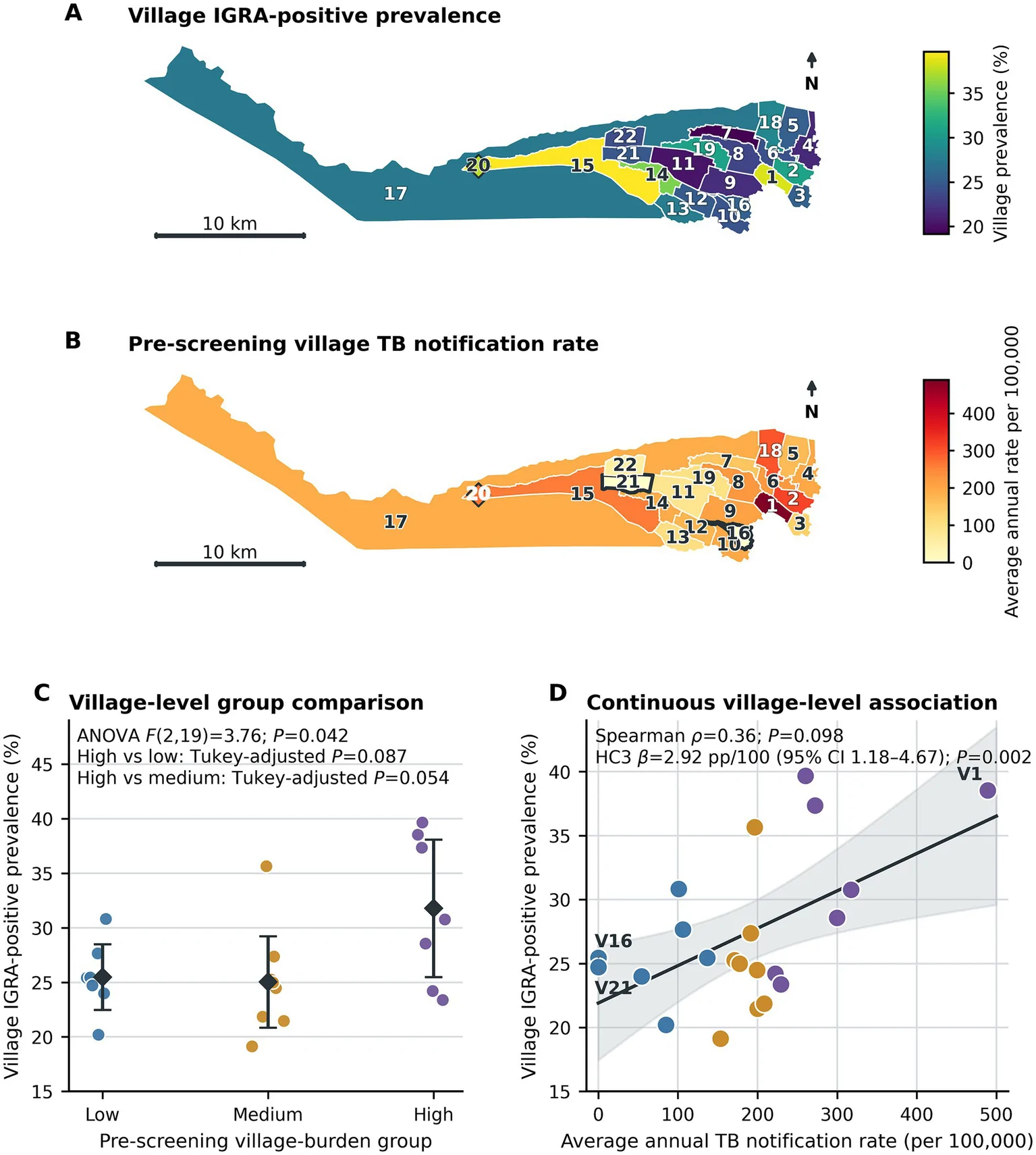
Geographic distribution of village-level IGRA positivity and pre-screening tuberculosis notification rates, with village-level analyses. **(A)** IGRA-positive prevalence and **(B)** archived average annual TB notification rate from January 2021 through June 2024. Because an up-to-date official vector layer reflecting the current village-boundary configuration was unavailable, the project-supplied Shufu County layer was used as the cartographic base, and village IDs and label positions were reconciled with the current village distribution and project administrative records. Distinct features in the available base layer represent 21 analytic units. Unit 20 is shown at its recorded location with a diamond because no distinct feature was available; no boundary was inferred. The map is a thematic representation of the observed village values, not the latest legal administrative-boundary map. Fill color represents the panel-specific value, and village IDs 1–22 are the only map labels. Villages 16 and 21, with confirmed zero notifications during the defined period, have bold outlines in panel B. **(C)** Village prevalence by tertile-defined burden group; points are villages, and diamonds with bars show unweighted means with 95% confidence intervals. **(D)** Average annual notification rate and village prevalence with an OLS fitted line and 95% confidence band. Only Villages 1, 16, and 21 are labeled in panel D. Spearman correlation was primary; the HC3 slope is reported as percentage points per 100 notifications per 100,000. All 22 units were retained. HC3, heteroscedasticity-consistent covariance estimator 3; IGRA, interferon-gamma release assay; OLS, ordinary least squares; TB, tuberculosis.

### AIMTB testing and outcome

Venous blood was collected for the *M. tuberculosis*-specific cellular immune response detection kit (AIMTB; Leide Biosciences Co., Ltd., Guangzhou, China; Chinese medical-device registration no. 20193400999). AIMTB is approved for clinical use in China. Samples were processed at the Shufu County Disease Prevention and Control Center according to the manufacturer’s instructions. In brief, blood was added to negative-control (N), TB-antigen (T), and positive-control (P) tubes, incubated at 37 °C for 18–22 h, centrifuged, and tested by fluorescence immunochromatography. Results were interpreted sequentially from N, T–N, and P–N concentrations; the mutually exclusive criteria are reproduced in Supplementary Table S2. In a Chinese comparative study, AIMTB showed 94.47% agreement with QuantiFERON-TB Gold Plus and a kappa of 0.84 (13).

The study outcome was a positive AIMTB result. We use the term ‘IGRA positivity’ because the assay measures immune sensitization and cannot by itself distinguish active TB, recent infection, remote infection, or future progression. The 12 indeterminate results were excluded before person-level analysis.

## Statistical analysis

### Descriptive and village-level analyses

Categorical variables were summarized as counts and percentages and continuous variables as means with standard deviations or medians with interquartile ranges. Wilson intervals were used for prevalence. Group comparisons used Pearson chi-square, Fisher exact, independent-samples *t*, Mann–Whitney *U*, one-way analysis of variance (ANOVA), or Kruskal–Wallis tests as appropriate. Age trend was evaluated with ordered age groups entered as one ordinal term.

Village-level IGRA prevalence was compared across burden groups with unweighted one-way ANOVA followed by all three Tukey–Kramer pairwise contrasts. Shapiro–Wilk tests assessed the marginal distributions of notification rate and village prevalence. Because village prevalence was non-normal, Spearman rank correlation was designated primary; Pearson correlation was retained as a supportive linear summary.

As an aggregate selection diagnostic, the number of unique adult participants in each village was divided by the corresponding all-age permanent-resident count. These descriptive proportions were compared across burden groups by one-way ANOVA and correlated with the continuous notification rate using Pearson and Spearman coefficients. Because the denominator was an all-age count rather than an adult-eligible count and the campaign was not probability sampled, the measure was interpreted only as program reach, not as an adult sampling fraction.

### Cluster-aware individual-level models

Both cluster-aware models used logistic generalized estimating equations (GEE), with the individual AIMTB result as the binary dependent variable (1 = IGRA positive; 0 = IGRA negative), a binomial variance function, and a logit link. Village defined the clustering unit, and an exchangeable working correlation was specified. Covariates were selected before model fitting from epidemiologic relevance and routine availability: sex, age group, lower-income program category, known TB exposure, and previous TB. The categorical GEE entered the three operational village-burden groups, with low burden as the reference; the continuous GEE entered the average annual notification rate scaled per 100 notifications per 100,000. Occupation was not modeled because 94.55% of participants worked in agricultural or pastoral production; education was added in a sensitivity model. Variance inflation factors were below 1.16.

With only 22 clusters, conventional sandwich standard errors can be too small. GEE coefficients were therefore paired with the Mancl–DeRouen bias-reduced sandwich covariance as the primary source of confidence intervals and Wald *p* values (14). As a separate sensitivity analysis, 22 villages were sampled with replacement in each of 1,000 cluster-bootstrap resamples; all participants within a sampled village were retained, repeated sampled clusters were relabeled, and the same exchangeable GEE was refitted. All 1,000 resamples converged. Percentile intervals used seed 20260813 for the categorical model and 20260814 for the continuous model.

Sensitivity models excluded participants with previous TB, excluded those with either previous TB or known TB exposure, and added education to the full cohort. Exploratory models were also fitted separately within each burden group. These within-group estimates were descriptive; no interaction test was specified, and sparse cells and residual clustering limit interpretation.

### Aggregate linear regression and diagnostics

The continuous village-level association was estimated by ordinary least squares (OLS) using heteroscedasticity-consistent covariance matrix estimator 3 (HC3) robust standard errors and expressed as the change in IGRA positivity rate in percentage points per 100 notifications per 100,000. Ramsey’s RESET, residual Shapiro–Wilk, and Breusch–Pagan tests assessed functional form, residual normality, and heteroscedasticity. Hat values and Cook’s distance were reviewed using 2*p*/*n* and 4/*n* screening thresholds. No village was removed. Tests were two-sided, and exact *p* values were reported to three decimals or as *p* < 0.001. Analyses were rerun from the frozen person-level data with reproducible code in Python 3.12.13 (pandas 2.2.3, NumPy 2.3.5, and SciPy 1.17.0).

### Ethics statement

The protocol was approved by the Ethics Committee of the Shufu County Disease Prevention and Control Center on 5 March 2024 (approval no. 2024-1). The study followed the Declaration of Helsinki. All analyzed participants provided written informed consent before study procedures.

## Results

### Analytic cohort

The IGRA file contained 3,756 adult test records before cleaning. Exclusion of 12 indeterminate results left 3,744 determinate records. Removal of two duplicate records after identifier reconciliation produced the frozen cohort of 3,742 unique adults: 1,006 IGRA positive and 2,736 IGRA negative. Overall positivity was 26.88% (95% CI 25.49–28.33%).

Relative to the available all-age permanent-resident count of 23,734, the 3,742 unique adult participants represented program reach of 15.77%; this descriptive proportion is not an adult sampling fraction (Supplementary Table S6).

### Participant characteristics

The cohort included 1,787 men and 1,955 women and had a mean age of 58.51 years (SD 11.14); 3,242 participants (86.64%) were aged 50 years or older. Most participants were Uygur (98.74%), and 94.55% worked in agricultural or pastoral production. IGRA positivity was 29.27% among men and 24.71% among women and increased across the ordered age groups. Positivity was also greater among participants in the lower-income program category, those reporting known TB exposure, and those with previous TB (Table 1; Figure 2). The estimates for participants with and without diabetes were 31.27 and 26.47%, respectively (*p* = 0.063).

**Table 1:** Characteristics of participating adults by IGRA result.

Characteristic	Total (*n* = 3,742)	IGRA positive (*n* = 1,006)	IGRA negative (*n* = 2,736)	*p* value
Sex, *n* (column %)				0.002
Men	1,787 (47.76)	523 (51.99)	1,264 (46.20)	
Women	1,955 (52.24)	483 (48.01)	1,472 (53.80)	
Ethnicity, *n* (column %)				0.125
Han	47 (1.26)	8 (0.80)	39 (1.43)	
Uygur	3,695 (98.74)	998 (99.20)	2,697 (98.57)	
Age, years, mean (SD)	58.51 (11.14)	60.36 (10.23)	57.84 (11.39)	<0.001
Education, *n* (column %)				<0.001
Primary school or lower	2,449 (65.45)	705 (70.08)	1,744 (63.74)	
Middle school	1,142 (30.52)	272 (27.04)	870 (31.80)	
High school or higher	151 (4.04)	29 (2.88)	122 (4.46)	
Occupation, *n* (column %)				<0.001
Administrative/institutional employees	162 (4.33)	23 (2.29)	139 (5.08)	
Commerce and service workers	42 (1.12)	9 (0.89)	33 (1.21)	
Agricultural/pastoral production	3,538 (94.55)	974 (96.82)	2,564 (93.71)	
Diabetes mellitus, *n* (column %)				0.063
Yes	323 (8.63)	101 (10.04)	222 (8.11)	
No	3,419 (91.37)	905 (89.96)	2,514 (91.89)	
Lower-income program category, *n* (column %)				<0.001
Yes	971 (25.95)	306 (30.42)	665 (24.31)	
No	2,771 (74.05)	700 (69.58)	2,071 (75.69)	
Known TB exposure, *n* (column %)				<0.001
Yes	111 (2.97)	45 (4.47)	66 (2.41)	
No	3,631 (97.03)	961 (95.53)	2,670 (97.59)	
Previous TB, *n* (column %)				<0.001
Yes	125 (3.34)	58 (5.77)	67 (2.45)	
No	3,617 (96.66)	948 (94.23)	2,669 (97.55)	

Values are *n* (column %) unless stated otherwise. *p* values are from Pearson chi-square tests for categorical variables and an independent-samples *t* test for age. IGRA, interferon-gamma release assay; SD, standard deviation.

**Figure 2:**
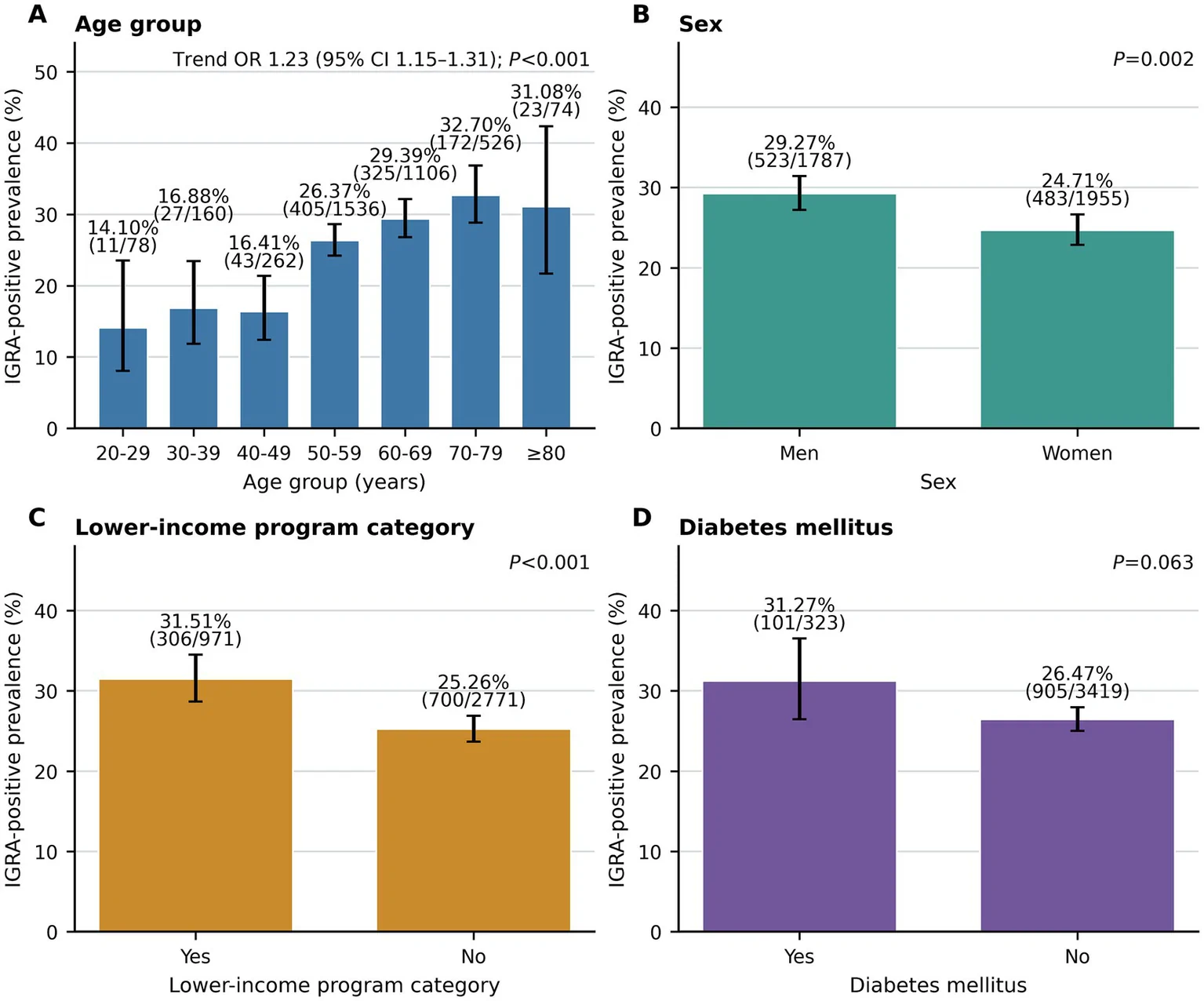
Prevalence of IGRA positivity by age and selected participant characteristics. **(A)** Age group, **(B)** sex, **(C)** lower-income program category, and **(D)** diabetes mellitus. Bars show prevalence with Wilson 95% confidence intervals; labels show the positive/total count. The age annotation reports the OR per ordered age-group increase. Other *p* values are from Pearson chi-square tests. CI, confidence interval; IGRA, interferon-gamma release assay; OR, odds ratio.

### Village distribution and aggregate analyses

Village-specific positivity ranged from 19.13 to 39.67%. The 22 units comprised seven low-, eight medium-, and seven high-burden villages. Pooled participant-level positivity was 24.51% (301/1,228; 95% CI 22.19–26.99%) in low-burden villages, 24.19% (307/1,269; 95% CI 21.92–26.62%) in medium-burden villages, and 31.97% (398/1,245; 95% CI 29.44–34.61%) in high-burden villages. High-burden villages contributed 33.27% of participants and 39.56% of all positive results. The number screened per positive result was 3.13 in high-burden villages, compared with 4.08 and 4.13 in low- and medium-burden villages (Figure 1; Supplementary Tables S4–S6). Thus, low- and medium-burden villages had nearly identical pooled positivity, whereas high-burden villages showed an absolute excess of approximately 7.5 percentage points and required about 23% fewer tests per positive result than low-burden villages.

Weighted program reach was 16.87, 15.43, and 15.13% in low-, medium-, and high-burden villages, respectively; village-specific values ranged from 11.05 to 22.72%. Program reach did not differ across burden groups (*F*(2, 19)=0.918; *p* = 0.416) and was not positively correlated with the notification rate (Pearson *r* = −0.386; *p* = 0.076; Supplementary Table S6). The higher positivity observed in high-burden villages was therefore not accompanied by greater relative program reach.

At village level, unweighted mean positivity was 25.47, 25.03, and 31.78% in the low-, medium-, and high-burden groups. The overall ANOVA gave *F*(2, 19)=3.76 (*p* = 0.042). The Tukey–Kramer-adjusted contrasts were −0.44 percentage points for medium versus low burden (*p* = 0.985), 6.31 points for high versus low burden (*p* = 0.087), and 6.75 points for high versus medium burden (*p* = 0.054). The overall difference numerically reflected higher mean positivity in high-burden villages, although neither adjusted pairwise comparison involving the high-burden group crossed the conventional 0.05 threshold.

Village positivity was non-normal (Shapiro–Wilk *p* = 0.027), so Spearman correlation was primary (*ρ* = 0.36; *p* = 0.098); Pearson correlation was *r* = 0.55 (*p* = 0.008). OLS diagnostics did not indicate material nonlinearity or heteroscedasticity. Village 1 had high leverage (hat = 0.410) but was not influential (Cook’s *D* = 0.127; screening threshold = 0.182) and remained in all analyses. Source surveillance records confirmed zero notifications for Villages 16 and 21 during January 2021–June 2024. The HC3-based OLS model estimated a 2.92-percentage-point higher village IGRA positivity rate for every increase of 100 notifications per 100,000 (95% CI 1.18–4.67; *p* = 0.002). The rank correlation did not reach the conventional significance threshold, whereas the linear model supported a positive rate–positivity gradient; this metric-dependent difference warrants cautious interpretation of the aggregate association.

### Cluster-aware individual-level analyses

In the categorical GEE, the adjusted OR was 1.369 for high- versus low-burden villages (95% CI 0.961–1.951; *p* = 0.082) and 0.979 for medium- versus low-burden villages (95% CI 0.759–1.262; *p* = 0.868). The village-bootstrap median OR for high versus low burden was 1.362 (95% percentile interval 1.011–1.921; Table 2; Supplementary Tables S7, S9). Thus, the high-versus-low estimate was directionally positive, but its precision depended on the small-sample method and remained less conclusive than the continuous-burden result.

**Table 2:** Adjusted associations with IGRA positivity from cluster-aware generalized estimating equation models.

Variable	Adjusted OR (95% CI)	*p* value	Village-bootstrap median OR (95% percentile interval)
Categorical operational model			
Sex			
Men	1.278 (1.081–1.511)	0.004	1.281 (1.095–1.512)
Women	Reference		Reference
Age group			
20–29	Reference		Reference
30–39	1.082 (0.547–2.140)	0.821	1.070 (0.579–2.232)
40–49	1.229 (0.449–3.361)	0.688	1.253 (0.512–2.887)
50–59	2.242 (1.210–4.153)	0.010	2.234 (1.296–4.324)
60–69	2.417 (1.276–4.580)	0.007	2.422 (1.402–4.714)
70–79	2.554 (1.278–5.105)	0.008	2.575 (1.416–5.168)
≥80	2.178 (0.831–5.709)	0.113	2.283 (0.845–5.865)
Lower-income program category			
Yes	1.244 (1.074–1.441)	0.004	1.245 (1.080–1.429)
No	Reference		Reference
Known TB exposure			
Yes	2.094 (1.620–2.706)	<0.001	2.093 (1.632–2.907)
No	Reference		Reference
Previous TB			
Yes	2.039 (1.443–2.882)	<0.001	2.029 (1.405–2.687)
No	Reference		Reference
Village-burden group			
Low	Reference		Reference
Medium	0.979 (0.759–1.262)	0.868	0.985 (0.785–1.240)
High	1.369 (0.961–1.951)	0.082	1.362 (1.011–1.921)
Continuous contextual model			
Average annual notification rate, per 100/100,000	1.153 (1.043–1.275)	0.006	1.152 (1.011–1.259)

Each model included sex, age group, lower-income program category, known TB exposure, and previous TB. The categorical model included burden-group indicators; the continuous model replaced those indicators with the pre-screening average annual notification rate. Odds ratios are exchangeable-GEE estimates. Confidence intervals and *p* values use the Mancl–DeRouen small-sample-corrected sandwich covariance for 22 village clusters. The sensitivity column reports 1,000 successful resamples of villages with replacement; all participants within a sampled village were retained. CI, confidence interval; GEE, generalized estimating equations; OR, odds ratio; TB, tuberculosis.

The continuous model gave an adjusted OR of 1.153 per 100 notifications per 100,000 (95% CI 1.043–1.275; *p* = 0.006); the village-bootstrap median OR was 1.152 (95% percentile interval 1.011–1.259). The corresponding estimates remained positive in the restricted and education-extended analyses (Supplementary Tables S8, S9), supporting the continuous notification rate as the more precise representation of village burden. In the categorical model, adjusted ORs were 1.278 for men (95% CI 1.081–1.511), 2.242 for ages 50–59 years (95% CI 1.210–4.153), 2.417 for ages 60–69 years (95% CI 1.276–4.580), 2.554 for ages 70–79 years (95% CI 1.278–5.105), 1.244 for the lower-income program category (95% CI 1.074–1.441), 2.094 for known TB exposure (95% CI 1.620–2.706), and 2.039 for previous TB (95% CI 1.443–2.882). The approximately twofold associations for ages 50–79 years, known TB exposure, and previous TB were stronger than those for male sex and the lower-income program category.

Exploratory within-burden models showed different patterns across the three panels of Figure 3. In low-burden villages (panel A), male sex, the lower-income program category, known TB exposure, and previous TB were associated with higher odds of IGRA positivity; the latter two estimates were approximately twofold, whereas age estimates were imprecise and did not show a consistent gradient. In medium-burden villages (panel B), ages 60–69 and 70–79 years were associated with higher odds, while the estimate for ages 50–59 years was similar in magnitude but its 95% CI included the null. In high-burden villages (panel C), male sex and previous TB were associated with higher odds; estimates for older age, the lower-income category, and known TB exposure were positive but imprecise. Because no interaction term was fitted, differences between panels should not be interpreted as evidence of effect modification.

**Figure 3:**
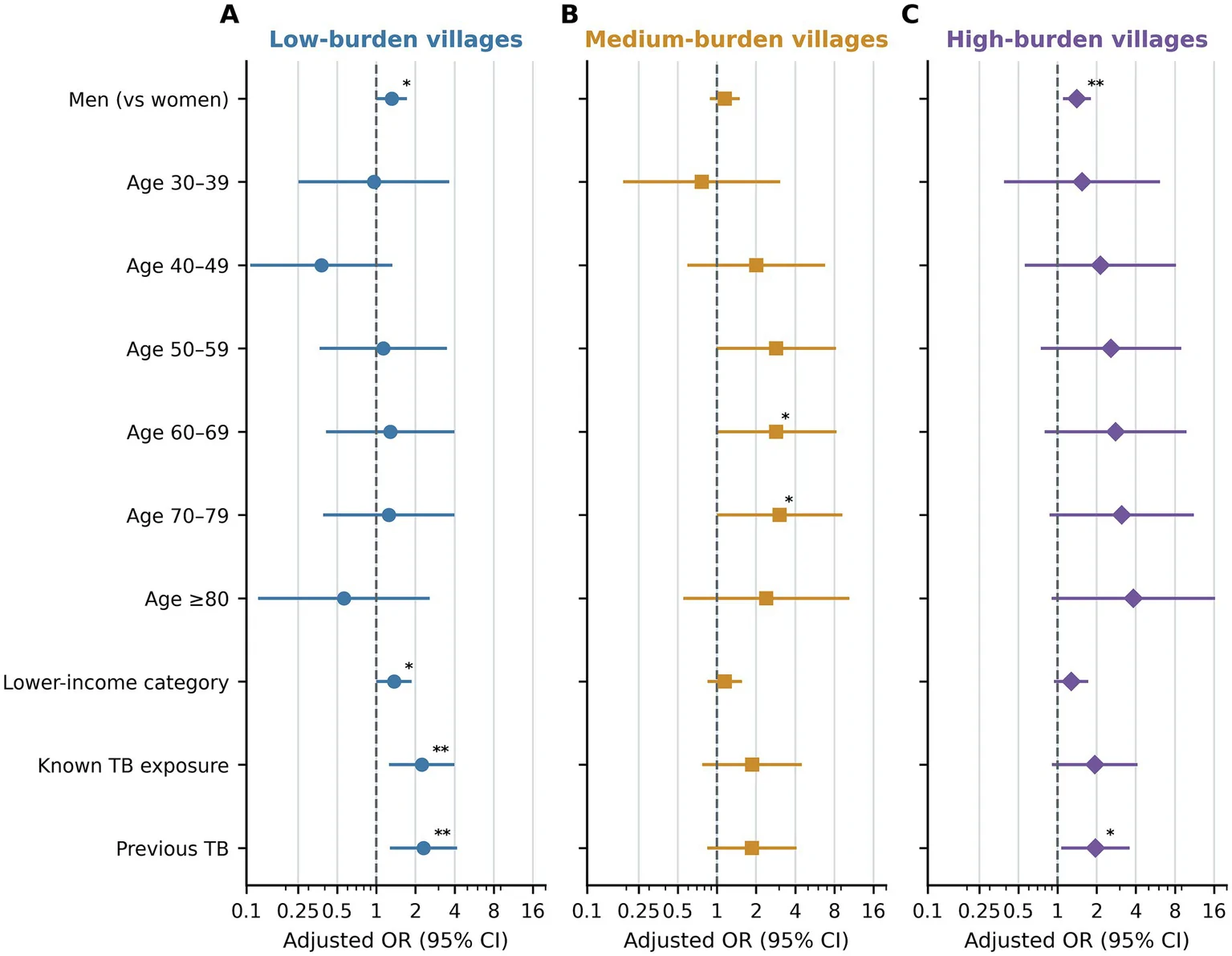
Exploratory adjusted associations with IGRA positivity within village groups defined by pre-screening tuberculosis notification rates. **(A)** Low-burden, **(B)** medium-burden, and **(C)** high-burden villages. Markers show adjusted odds ratios and horizontal lines show 95% confidence intervals from separate within-group models containing the variables displayed. Asterisks indicate two-sided Wald significance levels (^*^p < 0.05, ^**^p < 0.01, and ^***^p < 0.001). These analyses were exploratory; no interaction term was fitted, and sparse categories and residual within-village correlation limit interpretation. CI, confidence interval; IGRA, interferon-gamma release assay; OR, odds ratio; TB, tuberculosis.

## Discussion

Among the 3,742 participants, 26.88% were IGRA positive, and village estimates ranged from 19.13 to 39.67%. The central contextual finding was a rise in positivity with the pre-screening village notification rate in both the continuous individual-level GEE and the village-level linear model. High-burden villages yielded a larger share of positive results than of participants, although the adjusted tertile contrast was less precise. At participant level, male sex, ages 50–79 years, lower-income program category, known TB exposure, and previous TB were associated with higher positivity. Village surveillance history and routinely collected individual information therefore described different, complementary dimensions of testing yield.

The overall estimate is higher than the 13–20% QFT positivity reported across four population-based rural Chinese sites in 2013 (6), but lower than the 34.9% reported among older adults in a high-epidemic rural area of Zhejiang (15). Age structure is one likely contributor: 86.64% of this cohort was aged 50 years or older, and positivity rose from 14.10% at ages 20–29 years to more than 30% in the oldest groups. The setting also matters. Southern Xinjiang has sustained and geographically uneven TB notifications (11, 12), creating a different exposure history from that of many eastern Chinese cohorts. Sampling frame, calendar period, and assay procedures further limit direct comparisons. Even with those differences, finding more than one positive result for every four participants demonstrates the substantial pool of *M. tuberculosis* sensitization encountered by routine rural services and fills an important local evidence gap.

The way village burden was represented materially affected precision. Tertiles were easy to display, but they placed low- and medium-burden villages into groups with almost identical pooled positivity and discarded variation within each category. Retaining the original notification rate preserved that information. In the adjusted GEE, each additional 100 notifications per 100,000 was associated with 15.3% higher odds of IGRA positivity; the village-level model translated the same gradient into 2.92 percentage points of higher positivity. The cluster bootstrap reproduced the continuous GEE estimate. These concordant results across individual- and village-level scales support the continuous rate as the more informative measure for planning, while the three groups remain useful for communicating broad operational priorities.

The enrichment in the high-burden group is operationally relevant and was not accompanied by greater program reach in high-burden villages. These villages accounted for 39.56% of all positive results but 33.27% of participants, and 3.13 people were screened per positive result compared with 4.08 in low-burden villages. This corresponds to about 23% fewer tests per positive result in the high-burden group. This does not turn a notification rate into a diagnostic test; it shows that surveillance history can improve the order in which limited outreach and laboratory capacity are deployed. Universal access and contact investigation remain necessary because almost one quarter of participants in the low- and medium-burden groups were also positive.

Multi-year notification rates can mark communities with persistent concentrations of diagnosed disease and, therefore, greater opportunities for cumulative exposure. They may also reflect longer intervals before diagnosis, household and social mixing patterns, mobility, and socioeconomic conditions that shape contact with infectious TB. The association remained after adjustment for reported exposure and previous TB, suggesting that the village measure contains contextual information not fully captured by an individual questionnaire. At the same time, notification is a surveillance outcome: case detection, diagnostic access, reporting completeness, and denominator quality all contribute to its value. The confirmed zero values for Villages 16 and 21 therefore denote zero notified cases during January 2021–June 2024, not an absence of *M. tuberculosis* infection.

The participant-level associations add a second layer of information within each village. The age gradient is consistent with cumulative lifetime exposure and mirrors findings from rural Chinese cohorts (15); older adults also have a higher risk of developing TB disease (16). Higher positivity among men is consistent with the established sex imbalance in TB burden and may reflect differences in social contact, mobility, occupational exposure, and care seeking (17). Known TB exposure and previous TB each approximately doubled the adjusted odds of positivity and support prompt clinical review of these participants. The lower-income program category remained associated after adjustment and may capture housing, nutrition, mobility, and barriers to timely care that operate along the TB pathway (18).

AIMTB was deployed within the community campaign. The assay is approved for clinical use in China, can be processed locally by fluorescence immunochromatography, and showed 94.47% agreement with QuantiFERON-TB Gold Plus in a Chinese comparative study (13). As with other IGRAs, a positive result indicates immune sensitization to *M. tuberculosis* antigens; it does not date infection, distinguish remote from recent infection, or diagnose active disease (7, 19). That interpretation makes the established chest-radiography and referral pathway an essential companion to community infection testing.

IGRA testing is useful only when positive results lead to clinical assessment and appropriate follow-up. Active TB must be excluded before preventive treatment is considered (3), and treatment decisions must take account of clinical risk, contraindications, and participant preference (20). The prevention cascade can lose participants between testing, medical evaluation, treatment initiation, and completion (21, 22). In China, concerns about treatment can reduce acceptance (23), while stigma can affect both testing and treatment engagement (24). The annual examination platform can reduce some of these losses by bringing screening, chest radiography, referral, and follow-up into an existing service contact, but future implementation should measure each step of the cascade rather than treating IGRA positivity as the endpoint. Positivity was higher among participants with diabetes, but the difference was not statistically precise (*p* = 0.063). Diabetes remains relevant to clinical assessment because meta-analysis links it with TB infection (25), although it was not a central determinant in this study.

The findings suggest a feasible scheduling strategy within the existing platform. Before a campaign, villages can be ranked by the continuous notification rate and scheduled in phases. Mobile phlebotomy, incubation capacity, specimen transport, and clinical follow-up can then be concentrated early where the expected yield is greatest, while appointments remain available in every village. Community-based active case finding in China has shown the value of local delivery (26), and rural cohorts have documented ongoing infection and subsequent progression to disease (27, 28). Because IGRAs alone have limited positive predictive value for future TB (29, 30), village ranking should be combined with individual clinical assessment rather than used as a stand-alone eligibility rule.

Evidence from related implementation studies supports evaluation of this scheduling strategy. Modeling of spatially targeted chest-radiography screening suggests that adapting the sequence of community work to observed local yield can reduce the number of screens needed to find additional disease, while still requiring empirical evaluation (31). China’s preventive-treatment program expanded substantially during 2022–2025, but acceptance remains an important bottleneck and delivery through primary-care and designated facilities appears especially relevant (32). A recent Chinese prospective cohort also showed that short-course preventive treatment can be implemented with high completion among those who initiate therapy (33). The present study contributes the upstream evidence needed in southern Xinjiang: the size and distribution of the IGRA-positive pool, a reproducible village-level prioritization measure, and participant characteristics that can guide clinical follow-up. A prospective comparison of notification-guided and routine delivery should now assess diagnostic completion, preventive-treatment uptake and completion, incident TB, cost per completed preventive course, and equity across villages.

Several limitations define the scope of these conclusions. The cross-sectional design establishes association, not a causal effect of earlier village notifications on current IGRA positivity. Participation was voluntary, and individual-level data for adults who did not attend were unavailable; the 26.88% estimate therefore applies to campaign participants and should not be interpreted as township-wide adult prevalence. The village program-reach diagnostic used all-age resident denominators and cannot compare individual attendees with non-attendees, but participation did not increase with village notification burden, making differential village reach an unlikely explanation for the positive contextual association. Historical notification rates combine underlying disease burden with case detection and reporting performance. Inference was based on 22 village clusters, making the continuous estimate more stable than the tertile contrast. Finally, the available vector layer did not contain a distinct boundary for one study unit and did not represent the latest legal boundary configuration; the maps are therefore thematic displays rather than a basis for formal adjacency analysis. Despite these constraints, the individual- and village-level analyses gave consistent estimates of campaign yield and the continuous contextual association.

## Conclusion

More than one in four adults participating in the 2024 Zhanmin Township campaign were IGRA positive, revealing a substantial burden of *M. tuberculosis* sensitization in this high-burden rural setting. Higher pre-screening village notification rates consistently marked greater positivity in the continuous cluster-aware and village-level analyses, while male sex, ages 50–79 years, lower-income program category, known TB exposure, and previous TB identified additional high-yield groups. These findings provide direct epidemiologic support for combining village surveillance history with individual risk information to sequence IGRA outreach and clinical assessment within Xinjiang’s annual health-examination platform. They also establish a local baseline for planning and evaluating future tuberculosis preventive-treatment delivery in southern Xinjiang.

## Data Availability

The raw data supporting the conclusions of this article will be made available by the authors, without undue reservation.
