# Supplementary material for: Pre-screening village tuberculosis notification rates and the yield of interferon-gamma release assay testing in a rural health-examination campaign in Xinjiang, China
Source: Front Public Health. 2026 Sep 17;14:1869406. doi: 10.3389/fpubh.2026.1869406 (PMC13627292; doi:10.3389/fpubh.2026.1869406)
Supplement: Supplementary file 1 [file Supplementary_file_1.docx]

**Supplementary material**

**Supplementary Table 1. Data sources, variables, and operational definitions.**

| **Domain** | **Variable/source** | **Operational definition** |
| --- | --- | --- |
| Demographic | Sex, age, ethnicity | Annual examination record and structured questionnaire; age was analyzed in seven groups. |
| Socioeconomic | Education; occupation; lower-income program category | The lower-income category used the local threshold of household per capita annual income <RMB 5,292; continuous income was not collected. |
| Clinical | Diabetes mellitus | Self-report and routine examination record. |
| TB history | Known TB exposure; previous TB | Known TB exposure was a self-reported or program-recorded history of close contact with a person with TB before screening. Previous TB was a self-reported or program-recorded TB diagnosis before screening. |
| Village linkage | Village ID | Standardized 1–22 identifier used to link participant, notification, and boundary files; free-text address descriptors were not used for analytic linkage. |
| Contextual exposure | Archived average annual TB case-notification rate | Pre-calculated rate per 100,000 permanent residents for January 2021–June 2024, derived in the source surveillance file from notified cases and permanent-resident denominators; analyzed continuously and in data-derived tertiles. |
| Outcome | IGRA positivity | Positive AIMTB result under the sequential manufacturer criteria; 12 indeterminate results were excluded before analysis. |
| Program reach | Village participation proportion | Unique adult campaign participants divided by the all-age permanent-resident count; used only as an aggregate program-reach measure, not an adult sampling fraction. |

**Supplementary Table 2. Mutually exclusive AIMTB result-interpretation criteria.**

| **Sequential rule** | **Control condition** | **Additional condition** | **Interpretation** |
| --- | --- | --- | --- |
| 1 | N>400 pg/mL | Not applicable | Indeterminate |
| 2 | N≤400 pg/mL | T−N≥20 pg/mL and T−N≥25% of N | Positive |
| 3 | N≤400 pg/mL and positive rule not met | P−N≥25 pg/mL | Negative |
| 4 | N≤400 pg/mL and positive rule not met | P−N<25 pg/mL | Indeterminate |

Rules are applied from top to bottom so that every result has one interpretation. N, negative-control concentration; P, positive-control concentration; T, TB-antigen concentration. Twelve indeterminate results were excluded before the frozen analytic cohort was created.

**Supplementary Table 3. Participant selection and frozen IGRA analytic cohort.**

| **Stage** | **Denominator/status** | **Count** | **Interpretation** |
| --- | --- | --- | --- |
| Adult IGRA test records | Before analytic cleaning | 3,756 | July–August 2024 test export |
| Indeterminate IGRA results | Excluded | 12 | AIMTB rules in Supplementary Table 2 |
| Determinate test records | After indeterminate exclusion | 3,744 | Identifier and linkage checks applied |
| Duplicate identifier groups | Reconciled | 2 | One repeated record removed per group |
| Frozen analytic cohort | Unique adults | 3,742 | 22 villages/farm units |
| IGRA positive | Final cohort | 1,006 (26.88%) | Wilson 95% CI 25.49%–28.33% |
| IGRA negative | Final cohort | 2,736 (73.12%) | Determinate negative results |

Repeated identifiers were collapsed to one person-level record. Stable covariates agreed within each pair. One pair had concordant positive AIMTB results; the other had discordant valid positive and negative results and was classified positive under the project's deterministic any-positive reconciliation rule.

**Supplementary Table 4. Participant characteristics across village-burden groups.**

| **Characteristic** | **Total** | **Low (n=1,228)** | **Medium (n=1,269)** | **High (n=1,245)** | ***P* value** |
| --- | --- | --- | --- | --- | --- |
| **Sex** |  |  |  |  | **0.837** |
| Men | 1787 (47.76) | 578 (47.07) | 609 (47.99) | 600 (48.19) |  |
| Women | 1955 (52.24) | 650 (52.93) | 660 (52.01) | 645 (51.81) |  |
| **Ethnicity** |  |  |  |  | **0.022** |
| Han | 47 (1.26) | 18 (1.47) | 22 (1.73) | 7 (0.56) |  |
| Uygur | 3695 (98.74) | 1210 (98.53) | 1247 (98.27) | 1238 (99.44) |  |
| **Education** |  |  |  |  | **<0.001** |
| Primary school or lower | 2449 (65.45) | 722 (58.79) | 863 (68.01) | 864 (69.40) |  |
| Middle school | 1142 (30.52) | 465 (37.87) | 345 (27.19) | 332 (26.67) |  |
| High school or higher | 151 (4.04) | 41 (3.34) | 61 (4.81) | 49 (3.94) |  |
| **Occupation** |  |  |  |  | **0.411** |
| Administrative/institutional employees | 162 (4.33) | 51 (4.15) | 63 (4.96) | 48 (3.86) |  |
| Commerce and service workers | 42 (1.12) | 15 (1.22) | 10 (0.79) | 17 (1.37) |  |
| Agricultural/pastoral production | 3538 (94.55) | 1162 (94.63) | 1196 (94.25) | 1180 (94.78) |  |
| **Diabetes mellitus** |  |  |  |  | **0.546** |
| Yes | 323 (8.63) | 104 (8.47) | 103 (8.12) | 116 (9.32) |  |
| No | 3419 (91.37) | 1124 (91.53) | 1166 (91.88) | 1129 (90.68) |  |
| **Lower-income program category** |  |  |  |  | **0.007** |
| Yes | 971 (25.95) | 327 (26.63) | 359 (28.29) | 285 (22.89) |  |
| No | 2771 (74.05) | 901 (73.37) | 910 (71.71) | 960 (77.11) |  |
| **Known TB exposure** |  |  |  |  | **0.001** |
| Yes | 111 (2.97) | 54 (4.40) | 26 (2.05) | 31 (2.49) |  |
| No | 3631 (97.03) | 1174 (95.60) | 1243 (97.95) | 1214 (97.51) |  |
| **Previous TB** |  |  |  |  | **0.018** |
| Yes | 125 (3.34) | 51 (4.15) | 28 (2.21) | 46 (3.69) |  |
| No | 3617 (96.66) | 1177 (95.85) | 1241 (97.79) | 1199 (96.31) |  |
| Age, years, mean (SD) | 58.51 (11.14) | 58.58 (11.07) | 58.59 (11.30) | 58.37 (11.06) | 0.849 |

Values are n (column %) unless otherwise stated. *P* values are from Pearson chi-square tests for categorical variables and one-way ANOVA for age. SD, standard deviation; TB, tuberculosis.

**Supplementary Table 5. Village-level mapping and analytic values used in Figure 2.**

| **Village ID** | **Map representation** | **Average annual notification rate/100,000** | **n** | **Positive** | **Prevalence (%)** | **Group** |
| --- | --- | --- | --- | --- | --- | --- |
| 1 | Available base-layer feature | 489.07 | 96 | 37 | 38.54 | High |
| 2 | Available base-layer feature | 317.60 | 182 | 56 | 30.77 | High |
| 3 | Available base-layer feature | 136.70 | 228 | 58 | 25.44 | Low |
| 4 | Available base-layer feature | 200.00 | 219 | 47 | 21.46 | Medium |
| 5 | Available base-layer feature | 171.00 | 95 | 24 | 25.26 | Medium |
| 6 | Available base-layer feature | 222.42 | 157 | 38 | 24.20 | High |
| 7 | Available base-layer feature | 153.37 | 115 | 22 | 19.13 | Medium |
| 8 | Available base-layer feature | 229.23 | 231 | 54 | 23.38 | High |
| 9 | Available base-layer feature | 208.33 | 302 | 66 | 21.85 | Medium |
| 10 | Available base-layer feature | 199.32 | 147 | 36 | 24.49 | Medium |
| 11 | Available base-layer feature | 84.79 | 401 | 81 | 20.20 | Low |
| 12 | Available base-layer feature | 177.51 | 192 | 48 | 25.00 | Medium |
| 13 | Available base-layer feature | 106.04 | 188 | 52 | 27.66 | Low |
| 14 | Available base-layer feature | 196.19 | 115 | 41 | 35.65 | Medium |
| 15 | Available base-layer feature | 260.15 | 363 | 144 | 39.67 | High |
| 16 | Available base-layer feature | 0.00 | 110 | 28 | 25.45 | Low |
| 17 | Available base-layer feature | 191.42 | 84 | 23 | 27.38 | Medium |
| 18 | Available base-layer feature | 299.89 | 133 | 38 | 28.57 | High |
| 19 | Available base-layer feature | 100.81 | 133 | 41 | 30.83 | Low |
| 20 | Recorded location; no distinct polygon in supplied layer | 272.23 | 83 | 31 | 37.35 | High |
| 21 | Available base-layer feature | 0.00 | 93 | 23 | 24.73 | Low |
| 22 | Available base-layer feature | 54.35 | 75 | 18 | 24.00 | Low |

Only village IDs are printed in Figure 2. An up-to-date official vector layer reflecting the current village-boundary configuration was unavailable. Panels A and B therefore use the project-supplied Shufu County layer as a cartographic base; IDs and label positions were reconciled with the current village distribution and project administrative records. Distinct base-layer features represent 21 units. Unit 20 has no distinct feature and is shown at its recorded location with a diamond; no boundary was inferred. The display is thematic and does not represent the latest legal administrative boundaries. Fill color shows the mapped value. The archived notification rate covers January 2021–June 2024. Source surveillance records confirmed zero notifications for Villages 16 and 21 during this interval.

**Supplementary Table 6. Village-level comparisons, program-reach diagnostics, and aggregate linear model.**

| **Analysis** | **Effect/test** | **Estimate (95% CI where applicable)** | ***P* value/interpretation** |
| --- | --- | --- | --- |
| ANOVA | Overall burden-group difference | F(2,19)=3.76 | 0.042 |
| Tukey–Kramer | Medium vs low | -0.44 pp (-7.31 to 6.43) | 0.985 |
| Tukey–Kramer | High vs low | 6.31 pp (-0.78 to 13.40) | 0.087 |
| Tukey–Kramer | High vs medium | 6.75 pp (-0.12 to 13.62) | 0.054 |
| Shapiro–Wilk | Notification rate | W=0.952 | 0.341 |
| Shapiro–Wilk | Village prevalence | W=0.898 | 0.027 |
| Primary correlation | Spearman | ρ=0.36 | 0.098 |
| Supportive correlation | Pearson | r=0.55 | 0.008 |
| OLS diagnostic | Residual Shapiro–Wilk | W=0.959 | 0.465 |
| OLS diagnostic | Ramsey RESET | F=1.290 | 0.270 |
| OLS diagnostic | Breusch–Pagan | LM=0.382 | 0.536 |
| Influence | Village 1 | Hat=0.410; Cook's D=0.127 (4/n=0.182) | Not influential |
| HC3 OLS | Rate per 100/100,000 | β=2.92 pp (1.18 to 4.67) | 0.002 |
| Program reach | All 22 villages | 3,742/23,734=15.77%; village range 11.05%–22.72% | All-age denominator; not an adult sampling fraction |
| Program reach | Weighted low/medium/high | 16.87% / 15.43% / 15.13% | No increase with village burden |
| Participation diagnostic | ANOVA across burden groups | F(2,19)=0.918 | 0.416 |
| Participation diagnostic | Pearson: notification rate vs program reach | r=−0.386 | 0.076 |
| Participation diagnostic | Spearman: notification rate vs program reach | ρ=−0.286 | 0.196 |

Program reach was calculated using all-age permanent-resident counts and is presented only as an aggregate diagnostic of differential village participation; it is not an adult-eligible sampling fraction and does not compare individual attendees with non-attendees.

**Supplementary Table 7. Conventional and small-sample-corrected uncertainty for the categorical GEE.**

| **Term** | **Adjusted OR** | **Conventional robust 95% CI** | **Conventional *P*** | **Bias-reduced 95% CI** | **Bias-reduced *P*** |
| --- | --- | --- | --- | --- | --- |
| Men | 1.278 | 1.278 (1.089–1.500) | 0.003 | 1.278 (1.081–1.511) | 0.004 |
| Age 30–39 | 1.082 | 1.082 (0.565–2.073) | 0.812 | 1.082 (0.547–2.140) | 0.821 |
| Age 40–49 | 1.229 | 1.229 (0.506–2.987) | 0.649 | 1.229 (0.449–3.361) | 0.688 |
| Age 50–59 | 2.242 | 2.242 (1.235–4.070) | 0.008 | 2.242 (1.210–4.153) | 0.010 |
| Age 60–69 | 2.417 | 2.417 (1.306–4.474) | 0.005 | 2.417 (1.276–4.580) | 0.007 |
| Age 70–79 | 2.554 | 2.554 (1.308–4.985) | 0.006 | 2.554 (1.278–5.105) | 0.008 |
| Age ≥80 | 2.178 | 2.178 (0.884–5.370) | 0.091 | 2.178 (0.831–5.709) | 0.113 |
| Lower-income program category | 1.244 | 1.244 (1.080–1.432) | 0.002 | 1.244 (1.074–1.441) | 0.004 |
| **TB exposure history** | **2.094** | **2.094 (1.680–2.610)** | **<0.001** | **2.094 (1.620–2.706)** | **<0.001** |
| **Previous TB** | **2.039** | **2.039 (1.489–2.793)** | **<0.001** | **2.039 (1.443–2.882)** | **<0.001** |
| Medium vs low burden | 0.979 | 0.979 (0.782–1.224) | 0.851 | 0.979 (0.759–1.262) | 0.868 |
| High vs low burden | 1.369 | 1.369 (1.010–1.856) | 0.043 | 1.369 (0.961–1.951) | 0.082 |

Exchangeable working correlation=0.011; clusters=22. The bias-reduced covariance is primary. Core-model variance inflation factors were all <1.16.

**Supplementary Table 8. Sensitivity analyses for village notification burden.**

| **Analysis** | **n** | **Effect** | **Adjusted OR (bias-reduced 95% CI)** | ***P* value** |
| --- | --- | --- | --- | --- |
| Full cohort, categorical model | 3,742 | Medium vs low burden | 0.979 (0.759–1.262) | 0.868 |
| Full cohort, categorical model | 3,742 | High vs low burden | 1.369 (0.961–1.951) | 0.082 |
| Exclude previous TB | 3,617 | Medium vs low burden | 0.997 (0.772–1.287) | 0.981 |
| Exclude previous TB | 3,617 | High vs low burden | 1.400 (1.010–1.941) | 0.044 |
| Exclude previous TB or known exposure | 3,518 | Medium vs low burden | 1.013 (0.779–1.318) | 0.924 |
| Exclude previous TB or known exposure | 3,518 | High vs low burden | 1.412 (1.020–1.954) | 0.038 |
| Add education | 3,742 | Medium vs low burden | 0.975 (0.762–1.249) | 0.843 |
| Add education | 3,742 | High vs low burden | 1.361 (0.954–1.943) | 0.089 |
| Full cohort, continuous model | 3,742 | Per 100 notifications/100,000 | 1.153 (1.043–1.275) | 0.006 |

All models used exchangeable GEE with village clusters. The individual covariates were sex, age group, lower-income program category, known TB exposure, and previous TB where applicable. The education-extended model added education. CI, confidence interval; GEE, generalized estimating equations; OR, odds ratio; TB, tuberculosis.

**Supplementary Table 9. Village-cluster bootstrap settings and results.**

| **Model** | **Effect** | **Successful resamples** | **Failed fits** | **Seed** | **Median OR (95% percentile interval)** |
| --- | --- | --- | --- | --- | --- |
| Categorical GEE | High vs low burden | 1,000 | 0 | 20260813 | 1.362 (1.011–1.921) |
| Categorical GEE | Medium vs low burden | 1,000 | 0 | 20260813 | 0.985 (0.785–1.240) |
| Continuous GEE | Per 100 notifications/100,000 | 1,000 | 0 | 20260814 | 1.152 (1.011–1.259) |

Villages were sampled with replacement. All participants in a sampled village were retained, repeated sampled villages were relabeled, and the same exchangeable GEE was refitted. OR, odds ratio.

**Supplementary Figure 1. Participant selection for the adult IGRA analytic cohort.**

The July–August adult IGRA file contained 3,756 test records before analytic cleaning. Exclusion of 12 indeterminate AIMTB results left 3,744 determinate records. Deterministic identifier reconciliation reduced the file by two repeated records, yielding 3,742 unique adults: 1,006 IGRA positive and 2,736 IGRA negative.
